# *Aedes* larval bionomics and implications for dengue control in the paradigmatic Jaffna peninsula, northern Sri Lanka

**DOI:** 10.1186/s13071-021-04640-6

**Published:** 2021-03-18

**Authors:** Sinnathamby N. Surendran, Tibutius T. P. Jayadas, Vaikunthavasan Thiruchenthooran, Selvarajah Raveendran, Annathurai Tharsan, Sharanga Santhirasegaram, Kokila Sivabalakrishnan, Suthakar Karunakaran, Bharathy Ponnaiah, Laksiri Gomes, Gathsaurie N. Malavige, Ranjan Ramasamy

**Affiliations:** 1 Department of Zoology, University of Jaffna, Jaffna, Sri Lanka; 2 Department of Geography, University of Jaffna, Jaffna, Sri Lanka; 3 Centre for Dengue Research, University of Sri Jayewardenepura, Nugegoda, Sri Lanka

**Keywords:** *Aedes* larval ecology, *Aedes* larval indices, Anthropogenic environmental factors and dengue, Arboviral diseases, Jaffna peninsula, Salinity-tolerant *Aedes* vectors, Vertical dengue virus transmission

## Abstract

**Background:**

The larval bionomics of *Aedes* across the Jaffna peninsula in northern Sri Lanka was investigated to obtain information needed for developing more effective larval source reduction measures to control endemic arboviral diseases.

**Methods:**

The habitats of preimaginal stages of *Aedes* mosquitoes were surveyed, and ovitrap collections were carried out in densely populated areas of the Jaffna peninsula. *Aedes* larval productivities were analysed against habitat characteristics, rainfall and dengue incidence. Adults emerging from collected larvae were tested for dengue virus (DENV).

**Results:**

Only *Aedes aegypti*, *Ae. albopictus* and *Ae. vittatus* were identified in the field habitat collections and ovitraps. *Aedes aegypti* was the predominant species in both the field habitat and ovitrap collections, followed by *Ae. albopictus* and small numbers of *Ae. vittatus*. Tires and open drains were the preferred field habitats for *Ae. aegypti*, although larval productivity was higher in discarded plastic containers. The three *Aedes* species differed in field habitat preferences. Concomitant presence of the three *Aedes* species was observed in the field habitats and ovitraps. Larval productivities were inversely correlated with the salinity of the field habitat. Rainfall in the preceding month significantly correlated with larval productivity in the field habitats. DENV serotype 2 was detected in *Ae. aegypti* collected from ovitraps in the city of Jaffna. High Breteau, House and Container indices of 5.1, 5.1 and 7.9%, respectively, were observed in the field habitat surveys and ovitrap indices of up to 92% were found in Jaffna city.

**Conclusions:**

*Aedes* larval indices in populated areas of the peninsula showed a high potential for dengue epidemics. Unacceptable littering practices, failure to implement existing dengue control guidelines, vertical transmission of DENV in vector mosquitoes and preimaginal development in brackish water and open surface drains, as well as in domestic wells that provide potable water, are serious constraints to the current *Aedes* larval source reduction methods used to control dengue in the Jaffna peninsula. Similar shortcomings in arboviral disease control are likely present in other resource-constrained tropical coastal zones worldwide.

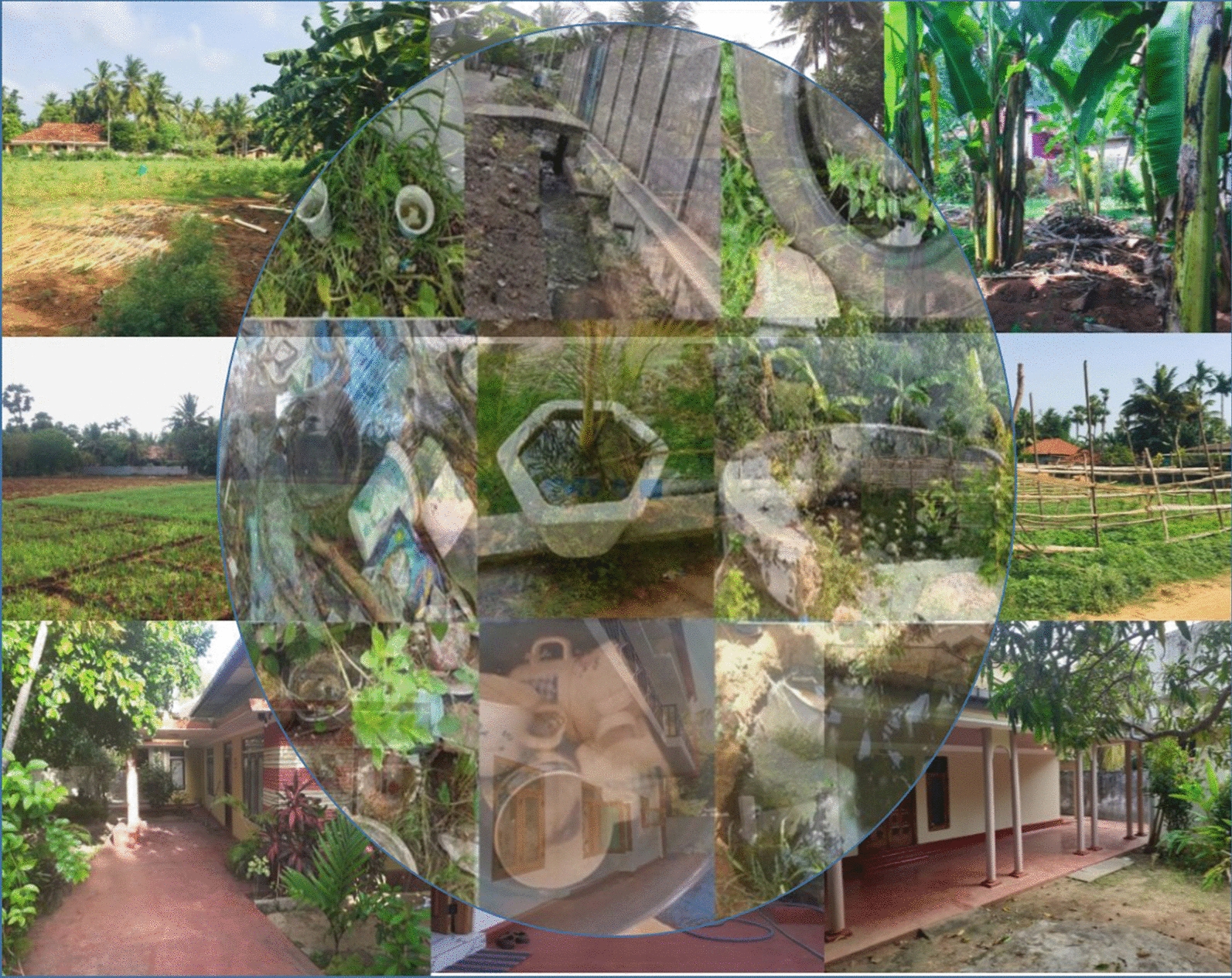

## Introduction

Dengue virus (DENV) is estimated to cause 390 (95% confidence interval: 284–528) million infections worldwide every year, with 96 million of these (95% confidence interval: 67–136) accompanied by clinical symptoms [1]. Approximately 70% of dengue infections occur in Asia [1]. *Aedes aegypti* and *Ae. albopictus* are the primary and secondary vectors, respectively, of dengue and are also vectors of other important arboviral diseases, such as chikungunya, yellow fever, Rift Valley fever and Zika worldwide [1–3]. Dengue has recently spread from the tropics to the temperate zone as a result of range expansion of the two vectors and the development of a cold-resistant, diapausing egg stage in *Ae. albopictus* [1]. The prevalence of dengue vectors, and hence the rate of dengue transmission, is influenced by many factors, including climate, human population density, the availability of habitats for preimaginal development in the human–environment and the vector control measures in use [1, 4, 5].

**Fig. 1:**
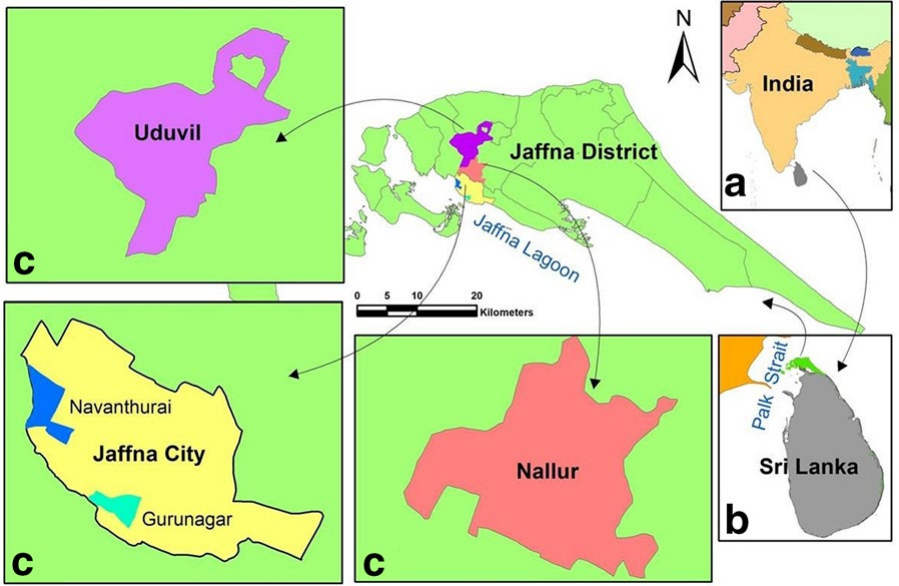
Map of the Jaffna peninsula in Sri Lanka in relation to India and showing the larval collection locations across the peninsula. **a** Location of Sri Lanka in relation to India, **b** location of Jaffna peninsula in northern Sri Lanka, **c** larval field collection locations in Nallur, Uduvil and Jaffna city (Gurunagar and Navanthurai wards in the city). Ovitrap collections were also conducted at several sites within Jaffna city

The Jaffna peninsula is located in northern Sri Lanka and is separated from the south Indian state of Tamil Nadu by the 64- to 137-km-wide Palk Strait (Fig. 1). The Jaffna lagoon lies between the peninsula and mainland Sri Lanka. The peninsula has a limestone geology and a maximum altitude of 10.4 m a.s.l., and it contains many small lagoons and seawater inlets. All locations in the peninsula are < 10 km from the sea, and therefore the entire peninsula can be considered to be a coastal zone. The Jaffna administrative district, which includes most of the peninsula and islands in the Palk Strait, has a land area of 1100 km^2^ and an average population density of approximately 700 persons/km^2^. Jaffna city is the largest and most populous urban center on the Jaffna peninsula, with an overall population density of 3048 persons/km^2^ and is the administrative capital of Jaffna district. The Jaffna peninsula has a tropical climate and receives much of its annual rainfall of 60–190 cm during the northeast monsoon that typically prevails from October through to January, with a variable, lower rainfall during the southwest monsoon from April through to June. The annual average temperature is 31.5 °C, and average relative humidity is 74.4%, with the temperature slightly decreasing and relative humidity increasing more markedly during the monsoon. The peninsula is undergoing rapid salinization of its groundwater due to the incursion of sea water as a result of unsustainable rates of water extraction from freshwater aquifers and rising sea levels [4, 6, 7]. Dengue has been present in Sri Lanka since the beginning of the 20th century, with a high prevalence in the Jaffna district in recent years; there have been approximately 200 reported cases of dengue per 100,000 persons in the district in 2019 [8, 9]. Chikungunya is the other main arboviral disease reported in the Jaffna district [10].

Features of the Jaffna peninsula make it a paradigmatic coastal zone for studying the impacts of population growth, anthropogenic environmental changes, global climate change and rising sea levels on mosquito-borne diseases [4, 11]. The preimaginal development of *Ae. aegypti* and *Ae. albopictus* in brackish water (BW) and freshwater (FW) habitats has been documented in the peninsula [8, 12–14], with FW, BW and saline water being defined as containing < 0.5 ppt (parts per thousand), 0.5–30 ppt and > 30 ppt salt, respectively [12].

The larval bionomics of anopheline mosquitoes in Jaffna city was recently described in the context of controlling a potential resumption of malaria transmission [15]. In contrast, only a very limited number of studies have been performed on the bionomics of *Aedes* vectors in the Jaffna peninsula [16, 17]. We therefore investigated the preimaginal habitats of *Aedes* species in areas of varying land use within the densely populated city of Jaffna and surrounding areas in 2018 and 2019 in relation to anthropogenic environmental factors, habitat characteristics, rainfall and dengue incidence. Ovitrap collections of larvae were also carried out at several sites within the city of Jaffna. We additionally investigated the presence of DENV in adult mosquitoes that emerged from the collected *Aedes* larvae.

## Methods

### Study areas

Potential larval habitats of *Aedes* species in the Jaffna peninsula were surveyed in Gurunagar (9°39′12.6″N, 80°01′03.5″E), Navanthurai (9°40′35.0″N, 80°00′12.4″E), Nallur (9°41′32.9″N, 80°01′22.7″E) and Uduvil (9°44′17.1″N, 80°00′37.0″E) (Fig. 1). Gurunagar and Navanthurai are municipal council wards within the city of Jaffna; Nallur is a suburban township bordering Jaffna city, with an average population density of approximately 1791 persons/km^2^; and Uduvil is a semi-agricultural township situated approximately 10 km to the north of Jaffna city, with an average population density of 1764 persons/km^2^. Ovitrap collections were made from 40 ovitraps placed at several locations within the city of Jaffna, including Gurunagar and Navanthurai.

### Field collection of larvae and larval indices

**Fig. 2:**
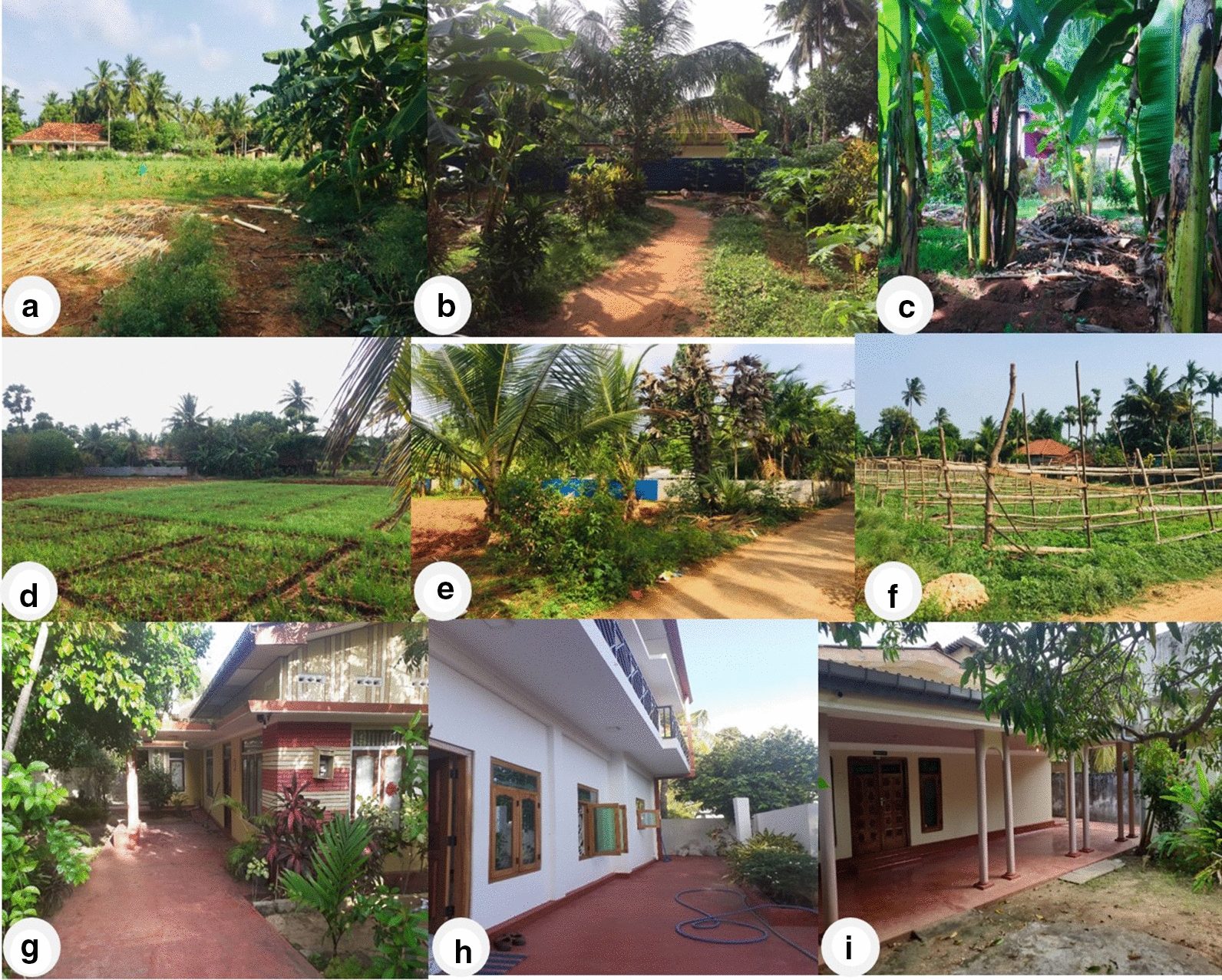
Examples of the environments (habitats) where field larval collections were made. **a**–**c** Small farms and gardens in suburban Nallur, **d**–**f** farmed areas in Uduvil, **g**–**i** houses with small gardens in Jaffna city

Surveys for *Aedes* larvae and larval collections were performed every 2 weeks from January 2018 to December 2019. Residential premises and a relatively smaller number of public and commercial premises and urban farms (for details, see Additional file 1) were randomly selected and then used throughout the study. Each location was inspected for water collection sites (termed habitats) suitable for the development of *Aedes* larvae (Fig. 2). Preimaginal stages were collected from habitats using a standard procedure consisting of ten dips with 350-ml dippers for water containers with wide openings (e.g. for drains and water storage tanks), pipetting with 5-ml plastic pipettes for smaller containers and ten dips with string-connected conical drop nets (diameter: 15 cm, depth: 10 cm) for wells, as described previously [15]. Sampling was performed in all selected potential habitats regardless of the presence or absence of preimaginal stages. Collected samples were linked to the habitat type and GPS location. The salinity of the habitat water was measured with a hand-held refracto-salinometer (Atago Co. Ltd., Tokyo, Japan). Collected preimaginal stages were brought to the laboratory of the Department of Zoology, University of Jaffna where pupae and dead preimaginal stages were discarded and live larvae were maintained under contained insectary conditions as previously described [16]. Adult mosquitoes emerging from collected larvae were identified to the species level with a standard key [18]. Larval productivity per month of each *Aedes* species in field habitats was determined as the cumulative number of adults emerging from the larvae collected each month from all the field habitats.

The Container Index (CI), House Index (HI) and Breteau Index (BI) for *Aedes* larvae were determined as recommended by the World Health Organization [19]. The preferences of *Aedes* species collectively and individually for the different types of habitats surveyed was assessed by calculating the habitat preference ratio (HPR). The overall HPR for all *Aedes* species was defined as the ratio of the proportion of each type of *Aedes* larva-positive habitat in all types of *Aedes* larva-positive habitats to the proportion of the same type of habitat among all types of surveyed habitats [20]. The HPR for individual *Aedes* species was calculated as the ratio of the proportion of each type of that *Aedes* species larva-positive habitat in all types of that *Aedes* species larva-positive habitats to the proportion of the same type of habitat among all types of surveyed habitats. The HPR indicates the preference for a particular type of habitat when varying numbers of different types of habitats are surveyed [20].

Associations between any two *Aedes* species in the field habitats were determined by 2 × 2 contingency tables for pairwise comparison [21].

### Ovitrap collections

**Fig. 3:**
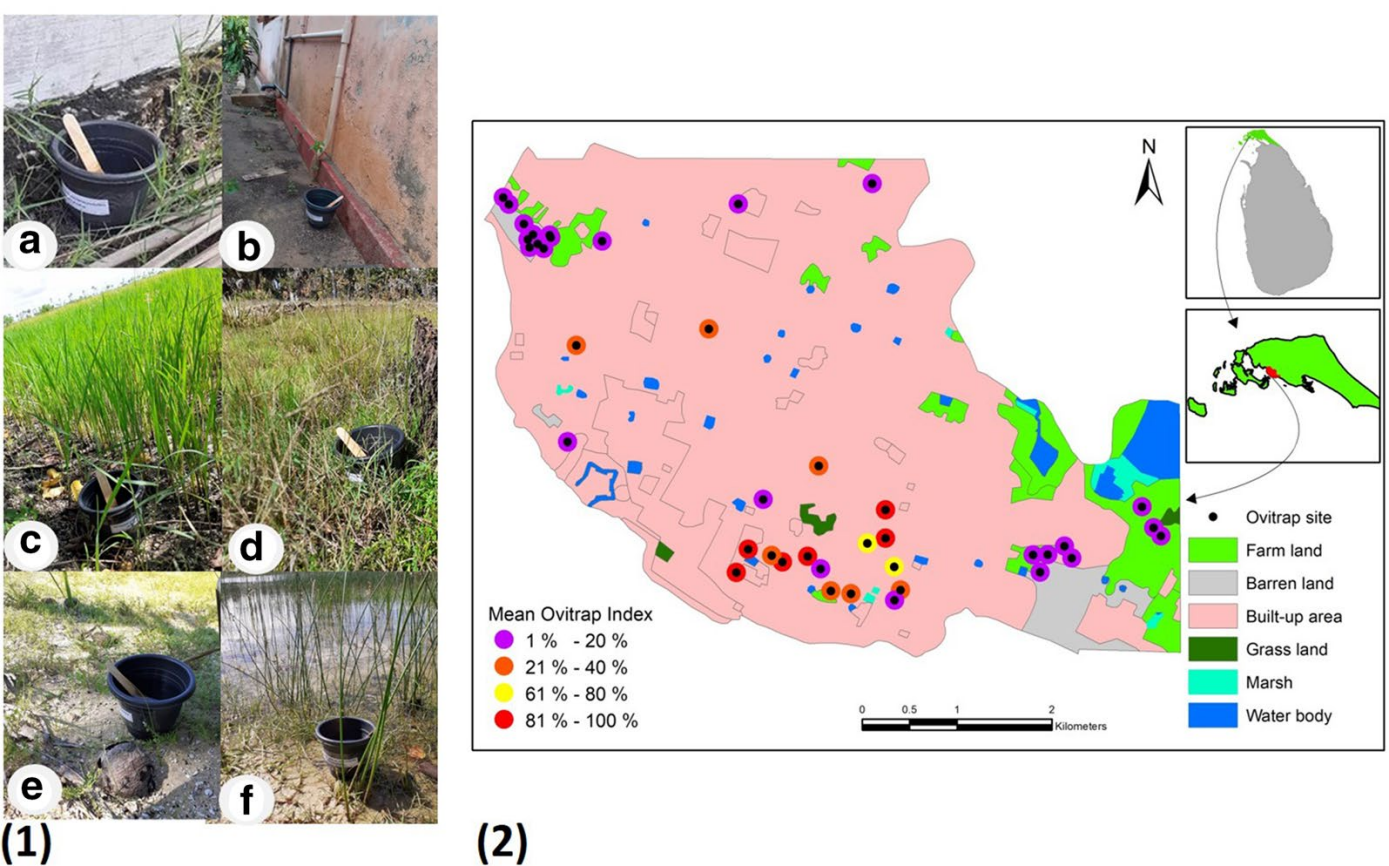
Ovitrap placement sites and their environment. **(1)** Ovitraps and placement sites: **a** black plastic ovitrap with plywood paddle resting against the inside rim, **b** built-up area, **c** farmland, **d** grassland, **e** barren land, **f** small lake. **2** Map of land use in Jaffna city, ovitrap placement sites and ovitrap indices

Conventional black plastic ovitraps (capacity: 650 ml, radius: 4.5 cm, height: 10 cm) containing 300 ml of water obtained from the nearest domestic water supply (i.e. well or tap) with a 2 × 15-cm plywood paddle resting against the inside upper rim, as shown in Fig. 3(1a), were utilized, as previously described [22]. The salinity of the habitat was measured with a hand-held refracto-salinometer (Atago Co. Ltd.). Forty outdoor ovitraps were placed at different locations in Jaffna city, with a minimum of distance of 15 m between ovitraps [Fig. 3(1b–f)], in areas of varying land use. Specifically, 20 ovitraps were located in built-up areas of the city, and the remaining 20 ovitraps were placed in the relatively limited number of areas in the city available for other types of land use, with nine ovitraps placed in selected sites in farmlands, five near water bodies, five on barren land and one on grassland [Fig. 3(2)].

Weekly collections were carried out from March 2019 to December 2019. After each collection of eggs and larvae at each site, ovitraps with a replacement paddle were filled with an equal amount of water from the same source as the preceding ovitrap for the next round of collection. Eggs and larvae from the ovitraps were brought to the laboratory and reared to adulthood. Emerging adults were identified to the species level [18]. The monthly ovitrap index (OI) for each of the 40 ovitraps was calculated as the percentage of the ratio of the number of times an ovitrap was positive for *Aedes* in the specific location to number of times the ovitrap was surveyed at the specific location. The average OI over the 9-month collection period was used for geospatial interpolation. Larval productivity per month of each *Aedes* species in the ovitraps was determined as the cumulative number of larvae produced each month from all the ovitraps.

### Statistical analysis of relationships between *Aedes* larval productivity, habitat characteristics, rainfall, environmental factors and dengue incidence

Monthly rainfall data for the Jaffna district were obtained from the Government Meteorological Department. Monthly dengue incidence reported for the Jaffna district was obtained from the Epidemiology Unit of the Ministry of Health.

The Pearson correlation coefficient analysis was performed to test relationships between rainfall, habitat salinity and dengue incidence with *Aedes* larval productivity in the different field habitats. This analysis was also performed with the exclusion of salinity for ovitrap collections. To determine the dominant *Aedes* species collected in field habitats and ovitraps, separate estimations were done using the generalized linear model followed by least-squares means. Statistical analyses were performed using the SAS University Edition (SAS Institute Inc., Cary, NC, USA). The threshold for statistical significance was set at *P* < 0.05.

### DENV nonstructural protein 1 antigen assay and serotyping

Adult *Aedes* mosquitoes emerging from larvae collected in field habitats and ovitraps, and subsequently identified to the species level, were screened for DENV, as described previously [8], in pools of 30–40 mosquitoes of each species. *Aedes* mosquitoes identified from field surveys were pooled based on location and type of habitat, and those from ovitrap surveys were pooled based on the different land-use environment. Each pool of mosquitoes was triturated in a 1.5-ml microfuge tube with a plastic disposable pestle for extracting soluble antigens in 300 µl of 0.01 M phosphate buffered saline (PBS) containing 1% v/v Triton X-100. The extract was then centrifuged at 10,000 *g* for 5 min, and  approximately  30 µl of supernatant tested for the DENV nonstructural protein 1 antigen (NS1Ag) using the Biocredit Dengue NS1Ag antigen test (RapiGen Inc., Gyeonggi-do, Republic of Korea).

Of the remaining supernatant from DENV NS1 antigen-positive extracts, approximately 140 µl was used for viral RNA extraction and DENV serotyping, as previously described [23]. Essentially, viral RNA was extracted using the QIAamp Viral RNA Mini Kit (Qiagen, Hilden, Germany) and transcribed to cDNA with the High Capacity cDNA Reverse Transcription Kit (Applied Biosystems, Foster City, CA, USA) according to the manufacturer’s protocol, in 20-µl reaction mixtures containing 1 × RT buffer, 1 × RT random primers, 10 × dNTPMix (100 mM), 50 U of MultiScribe™ Reverse Transcriptase, 20 U of RNase Inhibitor (Applied Biosystems), 10 µl of RNA and PCR-grade water (Applied Biosystems). The qPCR assay was performed using the TaqMan® Multiplex Master Mix (Applied Biosystems). The reaction mixture (total volume: 20 μl) contained: 1 × TaqMan multiplex master mix (containing Mustag Purple dye), 900 nM of each primer, 250 nM of each probe, 2 μl of cDNA and PCR-grade water (Applied Biosystems). Following an initial denaturation at 95 °C/20 s, the reaction was carried out at 95 °C/3 s and 60 °C/30 s for 40 cycles. The threshold cycle value (Ct) for each reaction was set manually. All assays were done in triplicate. After the primers and probes were validated, a multiplex method was optimized to quantify the four serotypes in a single reaction.

## Results

### *Aedes* larvae in field habitats

**Fig. 4:**
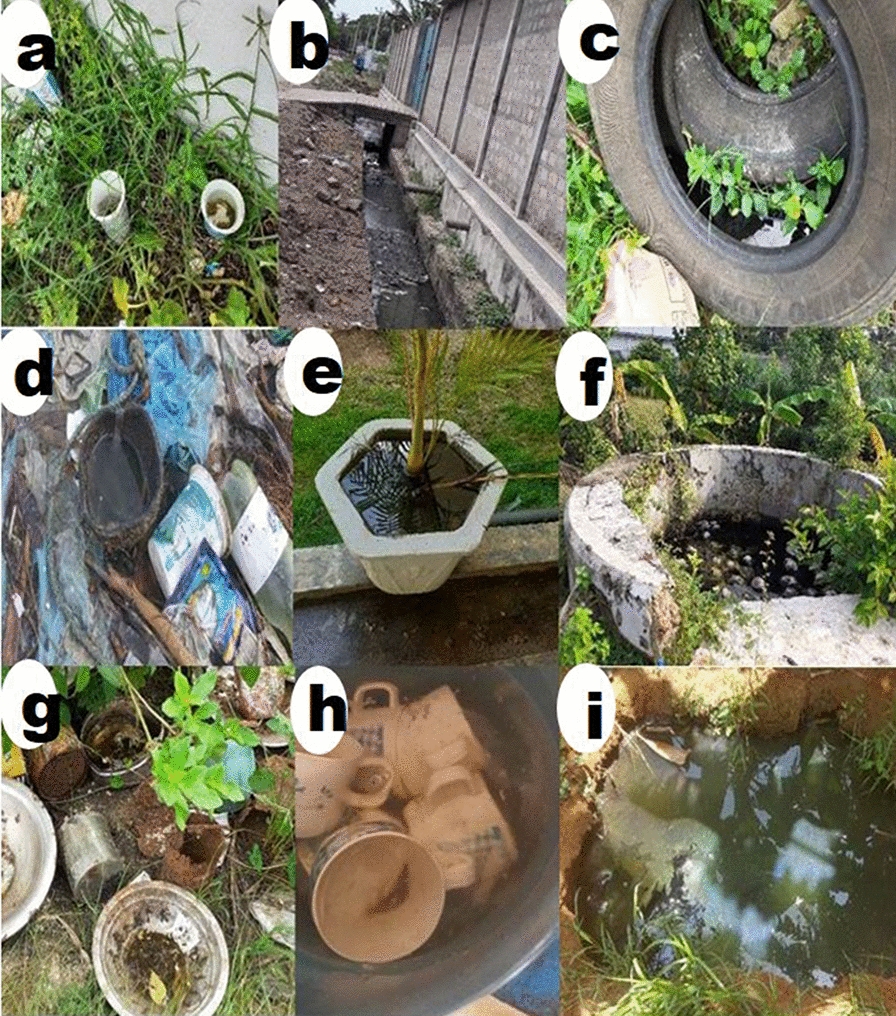
Examples of field habitats containing *Aedes* larvae. **a** Disposable plastic cups, **b** open surface drain, **c** discarded tire, **d** discarded coconut shell, **e** flower pot, **f** well, **g** discarded metal containers, **h** discarded ceramic containers, **i** water puddle

**Fig. 5:**
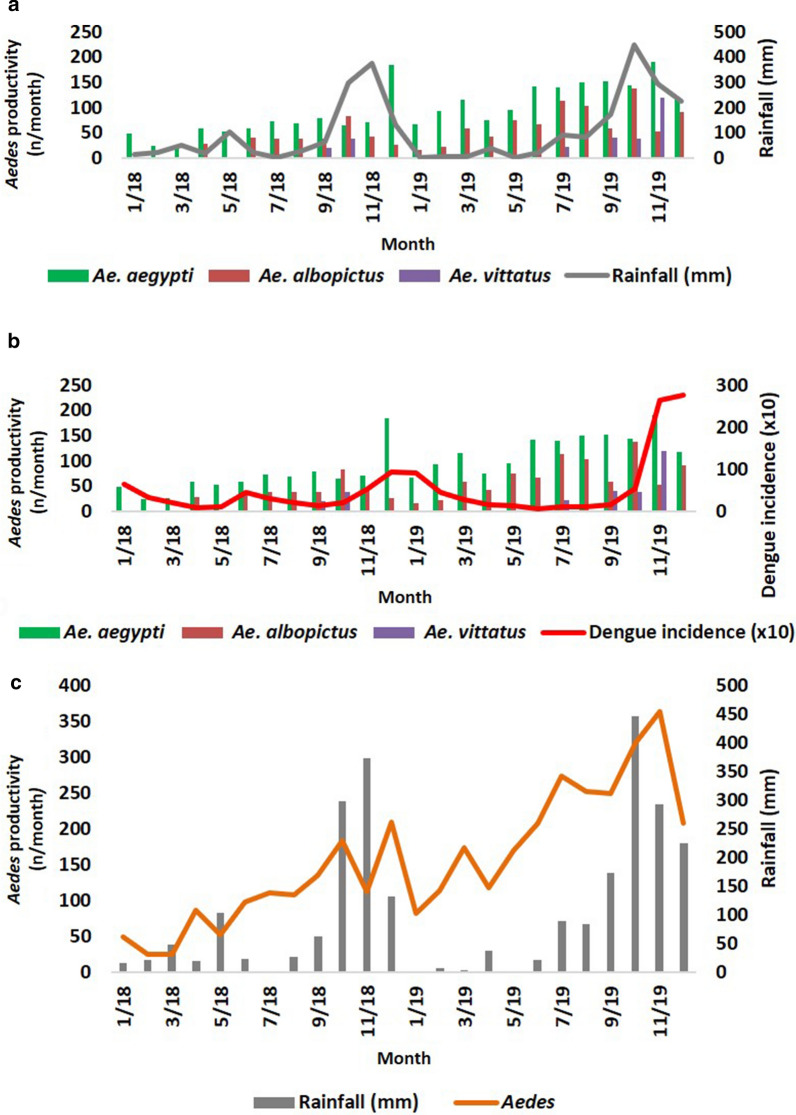
Monthly variation in the period January 2018 to December 2019 in larval productivity in the field habitats. Larval productivity of each *Aedes* species in the field habitats is shown in relation to rainfall (**a**) and dengue incidence (**b**), and as all *Aedes* species collectively in relation to rainfall (**c**)

A total of 3719 *Aedes* larvae of three *Aedes* species were collected from the different types of field habitats illustrated in Fig. 4. During the rainy season 1117 *Ae. aegypti*, 540 *Ae. albopictus* and 254 *Ae. vittatus* were collected, and during the dry season 1164 *Ae. aegypti*, 623 *Ae. albopictus* and 21 *Ae. vittatus* were collected; the monthly productivities are shown in Fig. 5a, b. Larval productivities were significantly different for each *Aedes* species throughout the study (*F*_(2, 33)_ = 21.52, *R*^2^ = 96.6, *P* < 0.001), with *Ae. aegypti* being the most abundant species with a mean (± standard deviation) monthly larval productivity of 97 ± 25.

Of a total of 2543 potential habitats examined during the field surveys, 202 were found to contain *Aedes* larvae. In these 202 habitats, the sole presence of either *Ae. aegypti*, *Ae. albopictus* or *Ae. vittaus* was observed in 133, 13 and three habitats respectively. The presence of larvae of more than one species was found in 53 habitats. A total of 2281, 1163 and 275 *Ae. aegypti*, *Ae. albopictus* and *Ae. vittaus*, respectively, were identified in several different types of habitats (Table 1). Discarded waste containers, particularly those made of plastic, were the most productive in terms of the number of *Aedes* larvae, accounting for 68% of all larval collections during the 2 years of survey. *Aedes aegypti* was the only species collected in open drains, for which it had the highest HPR of 2.5. Both *Ae. aegypti* and *Ae. albopictus* showed high HPRs of 2.2 and 6.3, respectively, for discarded tires. Although relatively fewer *Ae. vittatus* were collected, it showed the highest HPR of 20.1 for water puddles and was the only species collected in water puddles; both the BI and HI were 5.1% and the CI was 7.9% in the surveyed areas.

**Table 1:** *Aedes* larvae presence in different types of habitats

Type of habitat	Number of sampled habitats	Habitat salinity range (ppt salt)	Data for all *Aedes* species together			Data for each *Aedes* species separately								
						*Ae. aegypti*			*Ae. albopictus*			*Ae. vittatus*		
			No. of larvae collected	No. of positive habitats	HPR	No. of larvae collected	No. of positive habitats	HPR	No. of larvae collected	No. of positive habitats	HPR	No. of larvae collected	No. of positive habitats	HPR
Domestic wells	519	0–8	213	9	0.2	145	9	0.2	68	2	0.2	0	0	0.0
Open surface drains	194	0–3	390	35	2.3	390	35	2.5	0	0	0.0	0	0	0.0
Cement water storage tanks	235	0	383	14	0.8	144	11	0.7	162	7	1.2	77	2	2.0
Discarded plastic and paper cups	490	0	754	51	1.3	578	50	1.4	143	13	1.1	33	2	0.9
Discarded metal containers	338	0	83	6	0.2	83	6	0.2	0	0	0.0	0	0	0.0
Discarded plastic containers	515	0–5	1372	69	1.7	737	59	1.6	556	32	2.5	79	4	1.8
Discarded glass and ceramic containers	114	0	21	1	0.1	21	1	0.1	0	0	0.0	0	0	0.0
Discarded coconut shells	31	0	15	1	0.4	15	1	0.4	0	0	0.0	0	0	0.0
Flower pots and bases	40	0–8	149	6	1.9	60	4	1.4	63	3	3.0	26	1	5.8
Puddles	23	0	60	2	1.1	0	0	0.0	0	0	0.0	60	2	20.1
Discarded tires	44	0	279	8	2.3	108	7	2.2	171	7	6.3	0	0	0.0
Total (habitats or larvae)	2543		3719	202		2281	183		1163	64		275	11	

HPR, Habitat preference ratio; ppt, parts per thousand

*Aedes aegypti* was collected throughout the year, *Ae. albopictus* was collected more variably throughout the year and *Ae. vittatus* was collected mainly during the rainy season (Fig. 5a, b).

### Presence of larvae of different *Aedes* species in the same field habitat

*Aedes aegypti* was the most abundant species in the field collections, but the three species were sometimes present in the same or nearby habitats in the four areas studied (Additional file 1). *Aedes aegypti* and *Ae. albopictus* were found together in wells, cement water tanks, discarded plastic containers, flower pots/bases and tires; *Ae. aegypti* and *Ae. vittatus* were found together in discarded plastic containers; *Ae. albopictus* and *Ae. vittatus* were found together in flowerpots/bases. The concomitant presence of *Ae. aegypti* and *Ae. albopictus* was recorded in 25 and 20 habitats during the dry and rainy seasons, respectively. Larvae of all three species were found together in a single discarded plastic container during the wet season; however, Chi-square analysis for the concomitant presence of different species in the habitats (Additional file 2) suggested that *Ae. aegypti* and *Ae. albopictus* ($\chi_{{( 1,{ 2}0 2)}}^{ 2}$ = 47.69,* df * = 1, *P* < 0.01) as well as *Ae. aegypti* and *Ae. vittatus* ($\chi_{{( 1,{ 2}0 2)}}^{ 2}$ = 45.39,* df * = 1, *P* < 0.01) demonstrated significant development in different habitats. There was no statistical evidence to support the separate development of *Ae. albopictus* and *Ae. vittatus*.

### *Aedes* larval productivity in field habitats in relation to habitat salinity

The salinity in the habitats where *Aedes* larvae developed ranged between 0 ppt salt and 8 ppt salt (Table 1). Larval productivities of all three *Aedes* species combined (*r*_(24)_ = − 0.69, *P* = 0.0002) and *Ae. aegypti* alone (*r*_(21)_ = − 0.79, *P* < 0.0001) were significantly and negatively correlated with increasing salinity in the different habitats (Additional file 3).

### *Aedes* larval productivity in field habitats in relation to rainfall

The monthly larval productivities of *Ae. aegypti*,* Ae. albopictus* and *Ae. vittatus* alone or all three species collectively were not significantly related to rainfall during the same month (*r*_(24)_ = 0.11–0.43, *P* = 0.1–0.58; Additional file 3). However, as can be seen in Fig. 5c, there was a delay in the increase in larval productivity following the onset of rains. When rainfall in the preceding 1-month period was analysed in relation to the monthly larval productivity, only *Ae. aegypti* larval productivity showed a significant positive correlation with the preceding month’s rainfall (*r*_(23)_ = 0.42, *P* < 0.05).

### *Aedes* larval productivity in field habitats in relation to dengue incidence

As shown in Fig. 5b, there was a delay between the increase in larval productivities of *Aedes* in field habitats and the increase in dengue incidence. Statistical analysis of the larval productivities with the monthly incidence of dengue in the Jaffna district is shown in Additional file 4. The monthly larval productivity of *Ae. aegypti* showed a tendency to correlate with monthly dengue incidence (*r*_(24)_ = 0.38, *P* = 0.06), while that of *Ae. vittaus* showed a significant positive association with monthly dengue incidence (*r*_(24)_ = 0.48, *P* = 0.016). Larval productivities of all three *Aedes* species collectively tended to approach significance for an association with dengue incidence (*r*_(24)_ = 0.38, *P* = 0.06). However, there were significant positive associations between the larval productivities of *Ae. aegypti* (*r*_(20)_ = 0.52, *P* = 0.01) and collectively for all three *Aedes* species (*r*_(20)_ = 0.59, *P* = 0.005) when larval productivities for the preceding 2 months were separately tested against dengue incidence in any given month.

### *Aedes* larval productivities in ovitraps

Larval productivities in the ovitrap collections were significantly different between the three species, with *Ae. aegypti* being the dominant species (*F*_(2,27)_ = 51.05, *P* < 0.001). Different species could be collected from the same or relatively proximal ovitraps (Additional file 5). The OIs for individual ovitraps varied from 5 to 92% in built-up areas, from 0 to 13% in farmlands and from 0 to 33% near water bodies; they were 0% on grasslands and barren land [Fig. 3(2)].

**Fig. 6:**
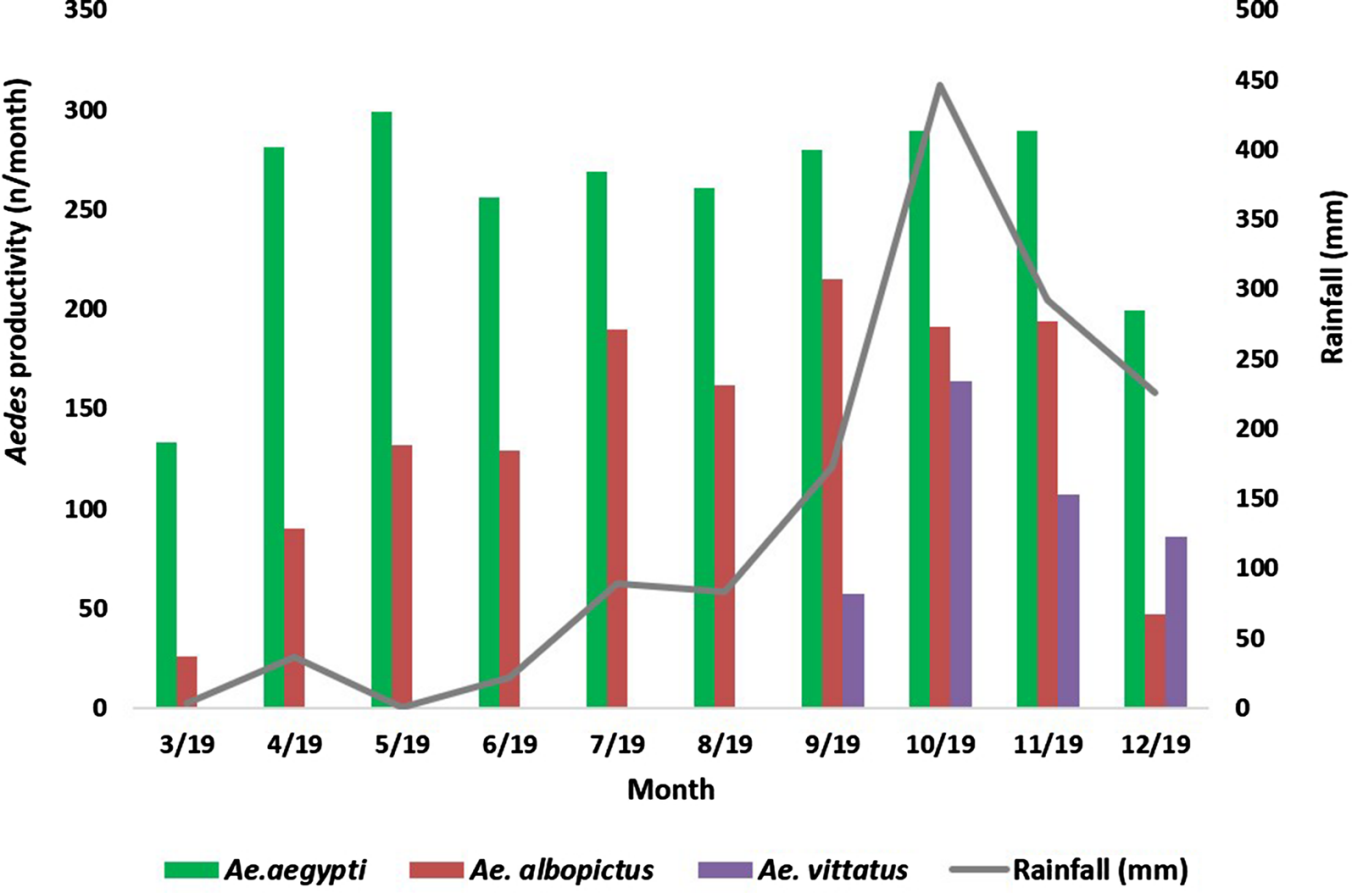
Monthly variation in larval productivity in ovitrap collections. Larval productivity of each *Aedes* species in ovitrap collections in relation to rainfall in the period from March 2019 to December 2019

Of the 40 ovitraps, the salinity of the water in six ovitraps was in the range of 1–3 ppt salt; all six of these ovitraps utilized water drawn from the nearest wells. All three species laid eggs and developed into preimaginal stages in a single ovitrap containing water with a salinity of 1 ppt salt. *Aedes aegypti* and *Ae. albopictus* laid eggs and developed into preimaginal stages in five ovitraps containing water with 1–3 ppt salt BW. Rainfall did not significantly influence the larval productivities of *Ae aegypti*, *Ae. albopictus* and collectively all larvae of all three species (*r*_(10)_ = 0.20–0.49, *P* = 0.14–0.57), with the exception of *Ae. vittatus* (*r*_(10)_ = 0.74, *P* = 0.01), which was only collected from ovitraps during the rainy season (Fig. 6).

### DENV NS1Ag test and DENV serotyping

Forty pools representing emergent adults of *Ae. aegypti* (21 pools), *Ae. albopictus* (15 pools) and *Ae. vittatus* (4 pools) collected from field habitats (8, 6 and 3 pools, respectively) and ovitraps (13, 9 and 1 pools, respectively) were separately tested for DENV NS1 antigen. A single pool of ovitrap-derived *Ae. aegypti* was found to be positive in the NS1Ag test. This pool of *Ae. aegypti* was collected in the month of October 2019 during the monsoon season, from a built-up area with the highest observed ovitrap index of 92% (Fig. 3(2)). It was shown to contain DENV-serotype 2 by reverse transcription-qPCR.

## Discussion

A previous survey of peridomestic mosquitoes during 1986 and 1987 detected only *Ae. aegypti*, *Ae. albopictus* and *Ae. novalbopictus* in Jaffna city [24]. Since 2011, however, *Ae. aegypti* and *Ae. albopictus* larvae have been collected from FW and BW field microhabitats of discarded food and beverage containers on beaches at concentrations of up to 15 and 14 ppt salt, respectively [11, 13], and in the water of domestic wells at up to 9 ppt salt located in the coastal areas of Jaffna peninsula [12]. *Aedes vittatus* is present in parts of mainland Sri Lanka [25, 26], including its largest city Colombo [27]. *Aedes vittatus* has been regarded as a sylvatic or peridomestic mosquito in rural environments, developing mainly in rock-holes [21, 28, 29]. Taken together, our data from Jaffna city and previous data from Colombo [29] suggest that *Ae. vittatus* has now adapted to develop in densely populated urban areas, showing a preference for water puddles over discarded waste containers in the limited number of collections carried out to date. Additional investigations to determine whether *Ae. vittatus* now demonstrates greater anthropophagic and anthropophilic behaviour in the Jaffna peninsula are justified. *Aedes vittatus* has been reported to be a competent vector of DENV, yellow fever virus and chikungunya virus [29, 30]. Our observation of a significant association between *Ae. vittatus* larval productivity and dengue incidence in the Jaffna peninsula suggests that further investigations are needed on *Ae. vittatus* as a potential vector of DENV in Sri Lanka.

Our observations show that the *Aedes* vectors are able to oviposit and undergo preimaginal development in a large variety of habitats in urban and semi-urban areas across the Jaffna peninsula. The habitats were associated with (i) domestic water storage and supply (i.e. open wells and cement water tanks); (ii) drainage (i.e. open surface drains); (iii) man-made litter (i.e. plastic, metal, glass/ceramic, rubber containers, coconut shells); (iv) rain-fed water puddles; (v) flower pots and their bases. A recent relevant study of urban farms in Miami-Dade County, Florida, USA, reported that storm drains, phytotelmata, discarded tyres and garbage cans contained significant numbers of *Ae. aegypti* [31]. Phytotelmata were not investigated in our study due to the limited availability of personnel for the study, but they are also likely to serve as habitats for *Aedes* in the Jaffna peninsula. High larval productivities were recorded in collections from the numerous discarded plastic containers. The data suggest varying preference for different types of habitats among the three *Aedes* species that warrants more detailed investigation than was possible in our study. Our findings suggest that *Aedes* vectors in the Jaffna peninsula are evolving to adapt to anthropogenic changes in the environment [5, 7]. In the Jaffna peninsula, *Ae. aegypti*, the urban vector of dengue, has become the dominant *Aedes* species in Jaffna city as well as in suburban Nallur and semi-rural Uduvil. Similarly, *Ae. albopictus*, typically regarded to be a peri-urban vector of dengue, has expanded its range to built-up areas of Jaffna city. Urban farms have been reported to provide favourable environments for *Aedes* mosquitoes in Florida, USA [31, 32], and this also appears to be the case in the Jaffna peninsula. A previous GIS-based study showed that the built-up areas of Jaffna city have a high local incidence of dengue [33], which is consistent with the present findings that show high ovitrap indices in the city. These factors have to be taken into consideration in developing appropriate vector control measures in the peninsula.

Our findings show that *Ae. aegypti*, *Ae. albopictus* and *Ae. vittatus* can be collected from field habitats and ovitraps, either the same ovitrap or ovitraps that are in relative proximity to each other, suggesting an overlap in the distribution of blood-fed female *Aedes* species in the studied areas. Interspecific larval competition between *Ae. aegypti* and *Ae. albopictus* and associated competitive population displacement reported in the field [34, 35] does not appear to be a significant factor at the locations studied in the Jaffna peninsula. The preimaginal stages of *Ae. aegypti* and *Ae. albopictus* are known to co-exist in the same water collection although one species may predominate [36–38]. However, our findings suggest that *Ae. aegypti* and *Ae. albopictus* show variations in preferences for different field habitats. We observed that *Ae. vittatus* showed a more marked temporal variation in larval abundance than either *Ae. aegypti* and *Ae. albopictus* in being collected mainly during the rainy season in both the field habitats and ovitraps. This temporal variation may be related to a lower density of *Ae. vittatus* in the peninsula that is particularly dependent on rainfall to expand populations, but more detailed investigations are needed to establish this.

The preimaginal habitats in our present study contained FW or BW of up to 8 ppt salt in field habitats and ovitraps, consistent with our previous finding of salinity-tolerant *Ae. aegypti* and *Ae. albopictus* in the Jaffna peninsula [12–14]. Anophelines prevalent in Sri Lanka have also been shown to develop in BW habitats in the Jaffna peninsula and along nearby coastal mainland areas, including the well-known malaria vectors *Anopheles culicifacies* [14, 39] and *An. stephensi* [15]. The entire human population of the peninsula relies on groundwater extracted from the aquifers to meet all water needs (e.g. drinking water, and water for domestic, agricultural and industrial needs), but this water is becoming increasingly brackish [4, 6, 7, 40–43]. The water in many domestic wells in the coastal areas nearby Jaffna city are brackish and yet support the preimaginal development of *Ae. aegypti* and *Ae. albopictus* [13, 14]. Heritable changes contribute to the greater salinity tolerance of *Ae. aegypti* in BW habitats in the peninsula [44, 45]. However, the inverse relationship between *Aedes* larval productivities and habitat salinity in field habitats suggests that the three *Aedes* species in the Jaffna peninsula retain a preference to oviposit in habitats of lower salinity. The tendency of BW-derived laboratory colonies of *Ae. aegypti* in the peninsula to prefer to oviposit in BW rather than FW in the laboratory [44] has therefore not yet spread widely into the wild populations of *Ae. aegypti* in the peninsula. However, the present findings reinforce our previous recommendations [7, 44] that dengue vector control measures in the Jaffna peninsula, which are currently only directed towards FW habitats, need to be extended to BW habitats.

The vertical or transovarial transmission of DENV to eggs, through larvae and then to emergent F1 generation adults in *Ae. aegypti* [46–48] and *Ae. albopictus* [49] is well documented. We previously observed extensive vertical transmission of DENV from infected blood-fed female *Ae. aegypti* and *Ae. albopictus* to larvae and adults of the F1 generation in the laboratory [8]. Vertical transmission may supplement more direct transmission of DENV through blood-feeding alone, but its role in dengue epidemiology needs to be more firmly established. Vertical transmission in dengue vectors that are able to develop in BW, which is not presently targeted in vector control programmes, may provide a reservoir of infected vectors that can maintain a basal level of DENV persistence in coastal areas during the dry season. This has been postulated to facilitate the increased transmission that occurs with the onset of the monsoon due to the rapid proliferation of vectors developing in FW in the Jaffna peninsula, a phenomenon that may be shared with similar locations worldwide [8].

The reduction of dengue vector larval sources in the Jaffna peninsula is an ongoing activity that is enforced with penalties by the Ministry of Health. Our findings from the field surveys showed a BI and HI of > 5% and a CI of 7.9%, with an OI of up to 92% in Jaffna city. These indices show that the current management approach has been ineffective in the Jaffna peninsula and also that there is a very high risk of dengue epidemics [50, 51], emphasizing the urgent need for more effective preventive measures. The present findings provide important information that is necessary for improving larval source reduction measures by: (i) identifying areas, habitats and times where and when these measures are likely to be most effective; (ii) pinpointing the need for improving waste management; (iii) highlighting the importance of extending control measures to BW habitats, surface drains and domestic wells; (iv) re-emphasizing the need to replace open surface drains with underground drains [16]; (v) reinforcing the need for using larvivorous fish in both BW and FW domestic wells that have previously been shown to effectively eliminate mosquito larvae in the Jaffna peninsula [15, 52, 53]; (vi) stressing the need for installing mosquito-proof coverings for domestic wells and water storage tanks; (vii) underscoring the need for improving surveillance and research on both adult and preimaginal stages of *Aedes* vectors.

## Conclusion

The two established arboviral vectors, *Ae. aegypti* and *Ae. albopictus*, and a potential vector, *Ae. vittatus*, develop in many different types of discarded containers near dwelling places, open surface drains and domestic wells, yielding high larval indices in populated areas of the Jaffna peninsula. The vertical (transovarial) transmission of DENV, salinity tolerance, preimaginal development in open surface drains, domestic wells that provide potable water and litter in the human–environment are particular challenges that need to be addressed. The findings, which may serve as a paradigm for many other tropical coastal locations, identify the urgent need to improve *Aedes* larval source reduction practices in order to control dengue and chikungunya in the Jaffna peninsula.

## Supplementary information

**Additional file 1: Table S1.** Annual trends of national emissions (% year-1) in the 28 European Union countries (EU-28) for sulfur oxides (SOx), nitrogen oxides (NOx), on-road transport NOx (NOx_road), non-methane volatile organic compounds (NMVOCs), ammonia (NH_3_), carbon monoxide (CO), particulate matter with an aerodynamic diameter lower than 2.5 μm and 10 μm (PM2.5 and PM10) over the time period 2000–2017. All trends are significant at *p* < 0.05 (Mann–Kendall). The increasing trends are in bold. **Table S2.** Minimum and maximum percentage of EU-28 population (in %) exposed to air pollutants concentrations (tropospheric ozone O_3_, nitrogen dioxides NO_2_, particulate matter PM2.5 and PM10) exceeding the European Union (EU) and World Health Organization Air Quality Guidelines (WHO AQG) limit or target values between 2000 and 2017. **Table S3.** Annual trends of mortality (number of deaths per 1,000,000 inhabitants per year) due to ambient particulate matter with an aerodynamic diameter lower than 2.5 μm (PM2.5) and tropospheric ozone (O_3_) over the time period 2000–2017 in the 28 European Union countries (EU-28) with associated significance level *p* (Mann Kendall ***p < 0.001; **p < 0.01; *p < 0.05; ^+^*p* < 0.1 and *p* > 0.1).

**Additional file 2.** *Aedes* species collections during field survey in Navanthurai (**a**), Gurunagar (**b**), Nallur (**c**) and Uduvil (**d**).

**Additional file 3.** Statistical analysis of the co-occurrence of different *Aedes* species in field habitats.

**Additional file 4.** Statistical analysis of the relationships between *Aedes* larval productivities in field habitats and rainfall, habitat salinity and dengue incidence.

**Additional file 5.** *Aedes* species collections in ovitraps in Jaffna city.

## Data Availability

All data generated during this study are included in this published article and its additional files. Voucher specimens of identified *Aedes* mosquitoes have been deposited in the Museum of the Department of Zoology, University of Jaffna.

## Abbreviations

BI: Breteau Index
BW: Brackish water
CI: Container Index
DENV: Dengue virus
FW: Freshwater
HI: House Index
HPR: Habitat preference ratio
NS1Ag: Nonstructural protein 1 antigen

## References

[1] World Health Organization. Dengue Fact Sheet. https://www.who.int/news-room/fact-sheets/detail/dengue-and-severe-dengue. Accessed 25 May 2020.

[2] Powell JR (2018). Mosquito-borne human viral diseases: why *Aedes aegypti*?. Am J Trop Med Hyg.

[3] Leta S, Beyene TJ, De Clercq EM, Amenu K, Kraemer MUG, Revie CW (2018). Global risk mapping for major diseases transmitted by *Aedes aegypti* and *Aedes albopictus*. Int J Infect Dis..

[4] Ramasamy R, Surendran S (2012). Global climate change and its potential impact on disease transmission by salinity-tolerant mosquito vectors in coastal zones. Front Physiol..

[5] Ramasamy R, Surendran SN (2016). Mosquito vectors developing in atypical anthropogenic habitats—global overview of recent observations, mechanisms and impact on disease transmission. J Vector Borne Dis..

[6] Uthayashangar S, Nanthakumaran A, Devaisy S (2019). Status of the saltwater intrusion in Jaffna, Sri Lanka. Ceylon J Sci..

[7] Surendran SN, Senthilnanthanan M, Jayadas TTP, Karunaratne SHPP, Ramasamy R (2020). Impact of salinization and pollution of groundwater on the adaptation of mosquito vectors in the Jaffna peninsula, Sri Lanka. Cey J Sci..

[8] Surendran SN, Veluppillai T, Eswaramohan T, Sivabalakrishnan K, Noordeen F, Ramasamy R (2018). Salinity tolerant *Aedes aegypti* and *Ae. albopictus*–infection with dengue virus and contribution to dengue transmission in a coastal peninsula. J Vector Borne Dis..

[9] Dengue Update. Epidemiology unit, Ministry of Health, Sri Lanka. http://www.epid.gov.lk/web/index.php?option=com_content&view=artic. Accessed 20 Jan 2020.

[10] Surendran SN, Kannathasan S, Kajatheepan A, Jude PJ (2007). Chikungunya-type fever outbreak: some aspects related to this new epidemic in Jaffna district, northern Sri Lanka. Trop Med Health..

[11] Ramasamy R, Surendran SN (2011). Possible impact of rising sea level on vector-borne infectious diseases. BMC Infect Dis.

[12] Ramasamy R, Surendran SN, Jude PJ, Dharshini S, Vinobaba M (2011). Larval development of *Aedes aegypti* and *Aedes albopictus* in peri-urban brackish water and its implications for transmission of arboviral diseases. PLOS Negl Trop Dis..

[13] Surendran SN, Jude PJ, Thabothiny V, Raveendran S, Ramasamy R (2012). Pre-imaginal development of *Aedes aegypti* in brackish and fresh water urban domestic wells in Sri Lanka. J Vector Ecol..

[14] Jude PJ, Tharmasegaram T, Sivasubramaniyam G, Senthilnanthanan M, Kannathasan S, Raveendran S (2012). Salinity-tolerant larvae of mosquito vectors in the tropical coast of Jaffna, Sri Lanka and the effect of salinity on the toxicity of *Bacillus thuringiensis* to *Aedes aegypti* larvae. Parasites Vectors..

[15] Surendran SN, Jayadas TTP, Tharsan A, Thiruchenthooran V, Santhirasegaram S, Sivabalakrishnan K (2020). Anopheline bionomics, insecticide resistance and transnational dispersion in the context of controlling a possible recurrence of malaria transmission in Jaffna city in northern Sri Lanka. Parasites Vectors..

[16] Surendran SN, Jayadas TTP, Sivabalakrishnan K, Santhirasegaram S, Karvannan K, Weerarathne T (2019). Development of the major arboviral vector *Aedes aegypti* in urban drain-water and associated pyrethroid insecticide resistance is a potential global health challenge. Parasites Vectors..

[17] Surendran SN, Kajatheepan A, Sanjeefkumar KFA, Jude PJ (2007). Seasonality and insecticide susceptibility of dengue vectors: an ovitrap based survey in a residential area in northern Sri Lanka. Southeast Asian J Trop Med Public Health.

[18] Mahadevan S, Cheong WH (1974). Chart to the identification of *Aedes* (Stegomyia) group and pictorial key to Mansonia (Mansonidae).

[19] World Health Organization (2003). Guidelines for dengue surveillance and mosquito control.

[20] Mukhtar M, Han Q, Liao C, Haq F, Arslan A, Bhatti A (2018). Seasonal distribution and container preference ratio of the dengue fever vector (*Aedes aegypti*, Diptera: Culicidae) in Rawalpindi, Pakistan. J Med Entomol..

[21] Hashim NA, Ahmad AH, Talib A, Athaillah F, Krishnan KT (2018). Co-breeding association of *Aedes albopictus* (Skuse) and *Aedes aegypti* (Linnaeus) (Dipera: Culicidae) in relation to location and container size. Trop Life Sci Res..

[22] Service MW (1976). Mosquito ecology. Field sampling methods.

[23] Jayathilaka D, Gomes L, Jeewandara C, Jayarathna G, Herath D, Perera P (2018). Role of NS1 antibodies in the pathogenesis of acute secondary dengue infection. Nat Commun..

[24] Rajendram GF, Antony NR (1991). Survey of peridomestic mosquito species of Jaffna peninsula in Sri Lanka. Southeast Asian J Trop Med Public Health.

[25] Barraud PJ (1934). Diptera vol. 5 family Culicidae Tribes Megarhinini and Culicini (fauna of British India, including Ceylon and Burma).

[26] Huang YM (1977). Medical antomology studies—VIII. Notes on the taxonomic status of *Aedes vittatus* (Diptera:Culicidae). Contrib Am Entomol Inst..

[27] Priyangika BAS, De Silva BGDNK, Jayatunga-Katuwawalage DPW, Wickramasinghe MB (2014). The association of environmental changes and the replacement of mosquito fauna in the Colombo District, Sri Lanka. J Trop For Environ..

[28] Diallo D, Sall AA, Diagne CT, Faye O, Faye O, Ba Y (2014). Zika virus emergence in mosquitoes in southeastern Senega1 2011. PLoS One.

[29] Sudeep A, Shil P (2017). *Aedes vittatus* (Bigot) mosquito: an emerging threat to public health. J Vector Borne Dis..

[30] Jupp PG, Mcintosh BM (1990). *Aedes furcifer* and other mosquitoes as vectors of chikungunya virus at Mica, northeastern Transvaal, South Africa. J Am Mosq Control Assoc..

[31] Wilke ABB, Carvajal A, Vasquez C, Petrie WD, Beier JC (2020). Urban farms in Miami-Dade County, Florida have favourable environments for vector mosquitoes. PLoS One.

[32] Wilke ABB, Vasquesz C, Carvajal A, Medina J, Chase C, Cardenas G (2020). Proliferation of *Aedes aegypti* in urban environments mediated by the availability of key aquatic habitats. Sci Rep..

[33] Kannathasan S, Antonyrajan A, Karunaweera N, Anno S, Surendran SN (2009). Identification of potential malaria risk areas of the Jaffna district of northern Sri Lanka: a GIS approach. J Natl Sci Found..

[34] Gilotra SK, Rozeboom LE, Bhattacharya NC (1967). Observations on possible competitive displacement between populations of *Aedes aegypti* Linnaeus and *Aedes albopictus* Skuse in Calcutta. Bull World Health Organization.

[35] Braks MH, Honorio NA, Lounibos LP, O-De-Oliveira L, Juliano AA (2004). Interspecific competition between two invasive species of container mosquitoes, *Aedes aegypti* and *Aedes albopictus* (Diptera: Culicidae), Brazil. Ann Entomol Soc Am..

[36] Paaijmans KP, Read AF, Thomas MB (2009). Understanding the link between malaria risk and climate. Proc Natl Acad Sci USA.

[37] Ho BC, Ewert A, Chew LM (1989). Interspecific competition among *Aedes aegypti*, *Ae. albopictus* and *Ae. triseriatus* (Diptera: Culicidae): larval development in mixed cultures. J Med Entomol.

[38] Lounibos LP, Bargielowski I, Carrasquilla MC, Nishimura N (2016). Coexistence of *Aedes aegypti* and *Aedes albopictus* (Diptera; culicidae) in Peninsula Florida two decades after competitive displacements. J Med Entomol.

[39] Jude PJ, Dharshini S, Vinobaba M, Surendran SN, Ramasamy R (2010). *Anopheles culicifacies* breeding in brackish waters in Sri Lanka and implications for malaria control. Malar J..

[40] Ramasamy R, Surendran SN, Jude PJ, Dharshini S, Vinobaba M, Morand S, Dujardin JP, Lefait-Robin R, Apiwathnasorn C (2015). Adaptation of mosquito vectors to salinity and its impact on mosquito-borne disease transmission in the South and Southeast Asian tropics. Socio-ecological dimensions of infectious diseases in Southeast Asia.

[41] Rajasooriyar LD, Mathavan V, Dharmagunewardene HA, Nandakumar V. Groundwater quality in the Valigamam region of the Jaffna Peninsula, Sri Lanka. In: Rivett MO, Davison RM, Hiscock KM, editors. Sustainable groundwater development. Special publications. London: Geological Society; 2002. p. 181–97.

[42] Joshua WD, Thushyanthy M, Nanthagoban N (2013). Seasonal variation of water table and groundwater quality of the karst aquifer of the Jaffna peninsula-Sri Lanka. J Natl Sci Found..

[43] Mikunthan T, Vithanage M, Pathmarajah S, Arasalingam S, Ariyaratne R, Manthrithilake H (2013). Hydrogeochemical characterization of Jaffna’s aquifer systems in Sri Lanka.

[44] Ramasamy R, Jude PJ, Veluppillai T, Eswaramohan T, Surendran SN (2014). Biological differences between brackish and fresh water-derived *Aedes aegypti* from two locations in the Jaffna Peninsula of Sri Lanka and the implications for arboviral disease transmission. PLoS One.

[45] Surendran SN, Sivabalakrishnan K, Jayadas TTP, Santhirasegaram S, Laheetharan A, Senthilnanthanan M (2018). Anal papillae changes in brackish water *Aedes aegypti*. J Vector Borne Dis..

[46] Khin MM, Than KA (1983). Transovarial transmission of dengue 2 virus by *Aedes aegypti* in nature. Am J Trop Med Hyg.

[47] Anjel B, Joshi V (2008). Distribution and seasonality of vertically transmitted dengue viruses in *Aedes* mosquitoes in arid and semi-arid areas of Rajasthan, India. J Vector Borne Dis..

[48] Arunachalam N, Tewari SC, Thenmozhli V, Rajendran R, Paramasivan R, Manavalan R (2008). Natural vertical transmission of dengue viruses by *Aedes aegypti* in Chennai, Tamil Nadu, India. Indian J Med Res.

[49] Kumari R, Kumar K, Chauhan LS (2011). First dengue virus detection in Aedes albopictus from Delhi, India. Its breeding ecology and role in dengue transmission. Trop Med Int Health..

[50] Udayanga NWBA, Aryaprema S, Gunathilaka HN, Iqbal M, Fernando T, Abeyewickreme W (2020). Larval indices of vector mosquitoes as predictors of dengue epidemics: an approach to manage dengue outbreaks based on entomological parameters in the districts of Colombo and Kandy, Sri Lanka. BioMed Res Int..

[51] Cheung KY, Fok MY (2009). Dengue vector surveillance and control in Hong Kong in 2008 and 2009. Dengue Bull..

[52] Surendran SN, Kajatheepan A, Jude PJ, Ramasamy R (2008). Use of tilapia, *Oreochromis mossambicus*, for the control of mosquito breeding in water storage tanks in the Jaffna district of Sri Lanka. Trop Med Health..

[53] Surendran SN, Sivabalakrishnan K, Sivasingham A, Jayadas TTP, Karvannan K, Santhirasegaram S (2019). Anthropogenic factors driving recent range expansion of the malaria vector *Anopheles stephensi*. Front Public Health.

