# Correction to: *Aedes* larval bionomics and implications for dengue control in the paradigmatic Jafna peninsula, northern Sri Lanka

**DOI:** 10.1186/s13071-021-04705-6

**Published:** 2021-04-21

**Authors:** Sinnathamby N. Surendran, Tibutius T. P. Jayadas, Vaikunthavasan Thiruchenthooran, Selvarajah Raveendran, Annathurai Tharsan, Sharanga Santhirasegaram, Kokila Sivabalakrishnan, Suthakar Karunakaran, Bharathy Ponnaiah, Laksiri Gomes, Gathsaurie N. Malavige, Ranjan Ramasamy

**Affiliations:** 1 Department of Zoology, University of Jaffna, Jaffna, Sri Lanka; 2 Department of Geography, University of Jaffna, Jaffna, Sri Lanka; 3 Centre for Dengue Research, University of Sri Jayewardenepura, Nugegoda, Sri Lanka

## Correction to: Parasites Vectors (2021) 14:162 https://doi.org/10.1186/s13071-021–04640-6

Following publication of the original article [1], it was noted that Additional file 1: Table S1 had been provided with the wrong caption. The correct caption of this file is as follows: Additional file S1: Details of premises selected for field surveys.

The original article has now been updated with the correct caption.

## References

[1] Surendran SN, Jayadas TTP, Thiruchenthooran V, Raveendran S, Tharsan A, Santhirasegaram S, Sivabalakrishnan K, Karunakaran S, Ponnaiah B, Gomes L, Malavige GN, Ramasamy R (2021). *Aedes* larval bionomics and implications for dengue control in the paradigmatic Jaffna peninsula, northern Sri Lanka. Parasites Vectors.

