# Supplementary figures and images for: Aedes larval bionomics and implications for dengue control in the paradigmatic Jaffna peninsula, northern Sri Lanka
Source: Parasit Vectors. 2021 Mar 18;14:162. doi: 10.1186/s13071-021-04640-6 (PMC7977581; doi:10.1186/s13071-021-04640-6)

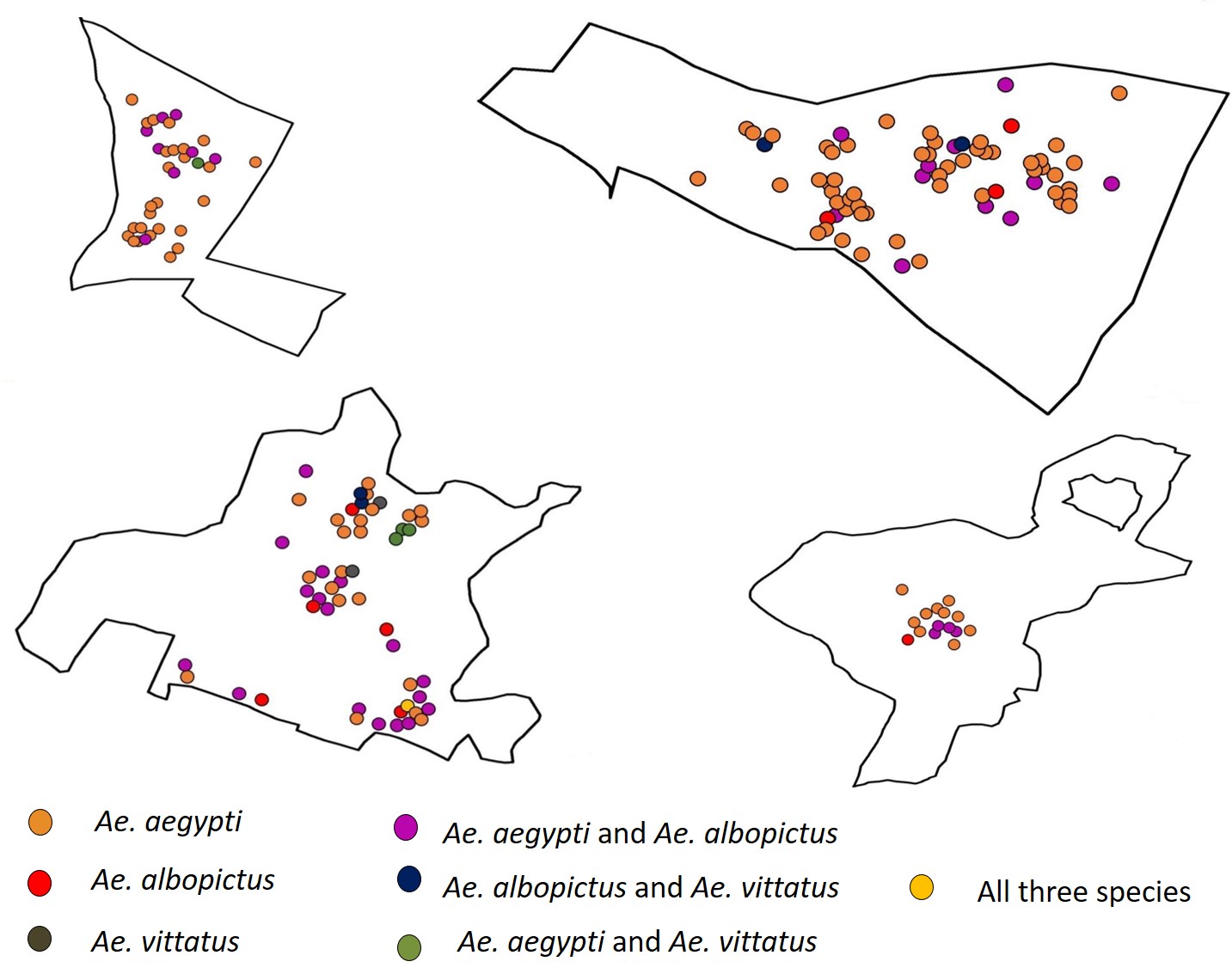

Supplement: Supplementary file 2 — Additional file 2. Aedes species collections during field survey in Navanthurai (a), Gurunagar (b), Nallur (c) and Uduvil (d). [file 13071_2021_4640_MOESM2_ESM.jpg]

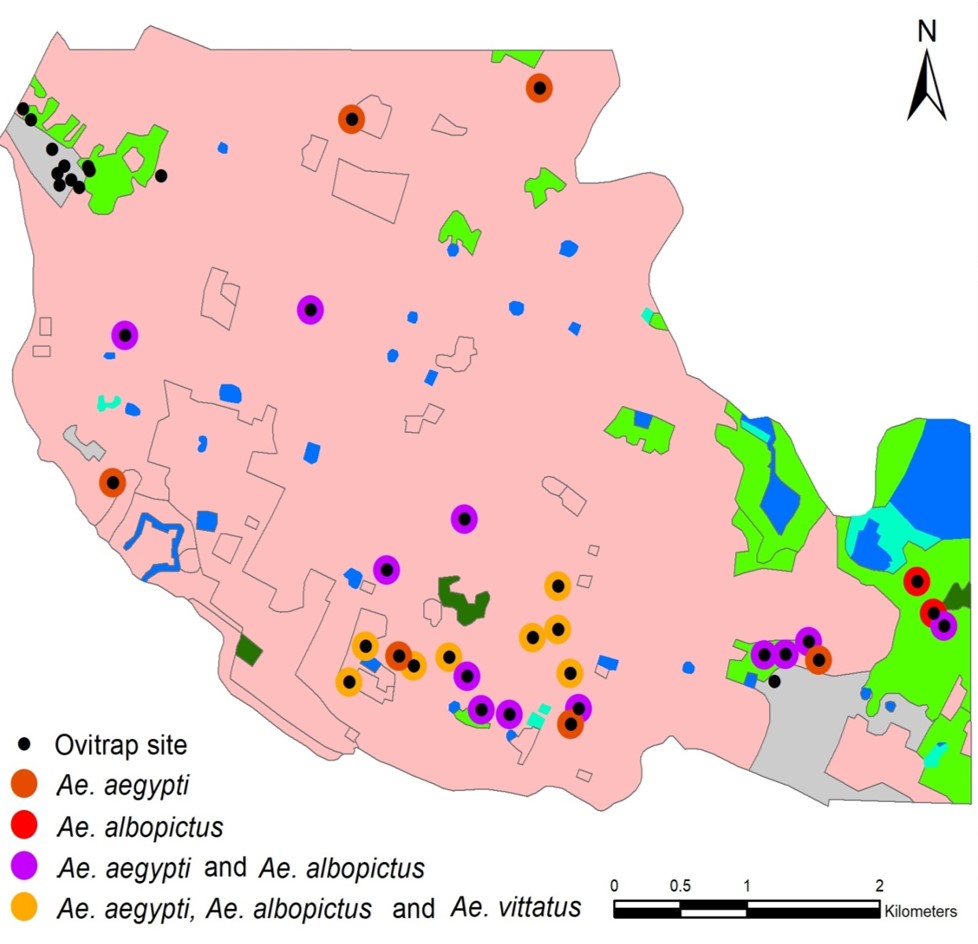

Supplement: Supplementary file 5 — Additional file 5. Aedes species collections in ovitraps in Jaffna city. [file 13071_2021_4640_MOESM5_ESM.jpg]
