# Supplementary material for: Aedes larval bionomics and implications for dengue control in the paradigmatic Jaffna peninsula, northern Sri Lanka
Source: Parasit Vectors. 2021 Mar 18;14:162. doi: 10.1186/s13071-021-04640-6 (PMC7977581; doi:10.1186/s13071-021-04640-6)
Supplement: Supplementary file 3 — Additional file 3. Statistical analysis of the co-occurrence of different Aedes species in field habitats. [file 13071_2021_4640_MOESM3_ESM.docx]

**Additional File S3.** Association between *Aedes* species in field habitats

**Table S3.1.** Association of *Aedes aegypti* and *Ae. albopictus* in field habitats

| *Ae. aegypti* (Species A) | | | | | |  |  |  |
| --- | --- | --- | --- | --- | --- | --- | --- | --- |
| Survey | Species B | | Presence | Absence | Total | ad-bc | X^2^ | P value |
| Field | *Ae. albopictus* | Presence | 47 a | 17 b | 64 | Negative | 47.69 | < 0.01 |
|  |  | Absence | 135 c | 3 d | 138 |  |  |  |
|  |  | Total | 182 | 20 | 202 |  |  |  |

**Table S3.2.** Association of *Ae. aegypti* and *Ae. vittatus* in field habitats

| *Ae. aegypti* (Species A) | | | | | |  |  |  |
| --- | --- | --- | --- | --- | --- | --- | --- | --- |
| Survey | Species B | | Presence | Absence | Total | ad-bc | X^2^ | P value |
| Field | *Ae. vittatus* | Presence | 3a | 6b | 9 | Negative | 45.39 | < 0.01 |
|  |  | Absence | 179c | 14d | 193 |  |  |  |
|  |  | Total | 182 | 20 | 202 |  |  |  |

**Table S3.3.** Association of *Ae. albopictus* and *Ae. vittatus* in field habitats

| *Ae. albopictus*  (Species A) | | | | | |  |  |  |
| --- | --- | --- | --- | --- | --- | --- | --- | --- |
| Survey | Species B | | Presence | Absence | Total | ad-bc | X^2^ | P value |
| Field | *Ae. vittatus* | Presence | 4 a | 3 b | 7 | Positive | 0.621 | 0.43 |
|  |  | Absence | 34 c | 40 d | 74 |  |  |  |
|  |  | Total | 38 | 43 | 81 |  |  |  |
