# Supplementary material for: Aedes larval bionomics and implications for dengue control in the paradigmatic Jaffna peninsula, northern Sri Lanka
Source: Parasit Vectors. 2021 Mar 18;14:162. doi: 10.1186/s13071-021-04640-6 (PMC7977581; doi:10.1186/s13071-021-04640-6)
Supplement: Supplementary file 4 — Additional file 4. Statistical analysis of the relationships between Aedes larval productivities in field habitats and rainfall, habitat salinity and dengue incidence. [file 13071_2021_4640_MOESM4_ESM.docx]

**Additional File S4.** Relationships between *Aedes* larval productivity in field habitats and rainfall, salinity and dengue incidence.

| **Parameter** | **Larval Productivity** | **N** | ***r*** | **Probability** |
| --- | --- | --- | --- | --- |
| Rainfall | *Ae. aegypti* | 24 | 0.11797 | 0.5830 |
|  | *Ae. albopictus* | 24 | 0.34319 | 0.1006 |
|  | *Ae. vittatus* | 24 | 0.26286 | 0.2146 |
|  | All *Aedes* | 24 | 0.27814 | 0.1882 |
| Salinity | *Ae. aegypti* | 21 | -0.79639 | <0.0001* |
|  | *Ae. albopictus* | 14 | -0.29986 | 0.2976 |
|  | *Ae. vittatus* | 5 | -0.83782 | 0.0765 |
|  | All *Aedes* | 24 | -0.69091 | 0.0002* |
| Dengue Incidence | *Ae. aegypti* | 24 | 0.38401 | 0.0639 |
|  | *Ae. albopictus* | 24 | 0.09790 | 0.6490 |
|  | *Ae. vittatus* | 24 | 0.48472 | 0.0164* |
|  | All *Aedes* | 24 | 0.38071 | 0.0665 |

**Legend.** * - Significant at P≤0.05; r – correlation coefficient; N – number of data points
